# Illuminating odors: when optogenetics brings to light unexpected olfactory abilities

**DOI:** 10.1101/lm.041269.115

**Published:** 2016-06

**Authors:** Julien Grimaud, Pierre-Marie Lledo

**Affiliations:** 1 Institut Pasteur, Laboratory for Perception and Memory, F-75015 Paris, France; 2 Centre National de la Recherche Scientifique (CNRS), Unité Mixte de Recherche (UMR) 3571, F-75015 Paris, France

## Abstract

For hundreds of years, the sense of smell has generated great interest in the world literature, oenologists, and perfume makers but less of scientists. Only recently this sensory modality has gained new attraction in neuroscience when original tools issued from physiology, anatomy, or molecular biology were available to decipher how the brain makes sense of olfactory cues. However, this move was promptly dampened by the difficulties of developing quantitative approaches to study the relationship between the physical characteristics of stimuli and the sensations they create. An upswing of olfactory investigations occurred when genetic tools could be used in combination with devices borrowed from the physics of light (a hybrid technique called optogenetics) to scrutinize the olfactory system and to provide greater physiological precision for studying olfactory-driven behaviors. This review aims to present the most recent studies that have used light to activate components of the olfactory pathway, such as olfactory receptor neurons, or neurons located further downstream, while leaving intact others brain circuits. With the use of optogenetics to unravel the mystery of olfaction, scientists have begun to disentangle how the brain makes sense of smells. In this review, we shall discuss how the brain recognizes odors, how it memorizes them, and how animals make decisions based on odorants they are capable of sensing. Although this review deals with olfaction, the role of light will be central throughout.

The media have considered many ways of exploring and modifying learning and memory processes, with more or less scientific accuracy (and popular success). Some of them, like *Eternal Sunshine of the Spotless Mind*, propose to erase specific memories by destroying the corresponding brain areas. Others suggest reprograming your personality with an inexpensive setup and some electric arcs above your head—the series *Dollhouse* is a prototypical example of these fantasies.

If all these movies appear eccentric, one of them was visionary. In 1997, secret U.S. agents also known as the *Men In Black* used a flash of light to erase short-term memory; this movie was released 2 yr before Francis Crick proposed himself that light could control the activity of specific neuron subtypes (Crick 1999). In 2002, the concept formulated by Francis Crick became a reality, when Gero Miesenbock's laboratory succeeded in exciting *Drosophila* neurons with flashes of light through the expression of a phototransduction cascade involving three proteins (Zemelman et al. 2002). This idea has now been developed into a technique routinely used in the field of neuroscience, namely optogenetics.

Optogenetics is based on the forced expression of a photosensitive channel (i.e*.*, opsin) on the surface of a specific neuron population. When flashed with light at a specific wavelength, such a channel causes a change in the membrane potential of the targeted neuron population (Goshen 2014). Various photosensitive channels have been developed with different electrical characteristics or response wavelengths, although most of them derive from opsins. The most commonly used opsin is called channelrhodopsin (ChR2), a light-activated cation-selective channel isolated from green algae called *Chlamydomonas reinhardtii* discovered in 2002 by the team of John Spudich (Sineshchekov et al. 2002), and first used to excite neurons 3 yr later by Boyden et al. (2005). When hit with blue light, the channel opens up, depolarizing the neural membrane and generating action potentials. In essence, the light incites the neuron to fire. In the same fashion, some opsins like halorhodopsins can inhibit neuron activity when they receive flash of lights (Guru et al. 2005).

Optogenetics has two major benefits. First, light allows a very fine and rapid control over the stimulation: in other words, it offers precise temporal control. Second, many genetic strategies have been developed to target specific cell types. Transgenic animals may carry opsin genes under the control of a specific promoter. Opsin genes may also be packed into a viral vector. Cell-type specificity may therefore be the result of a specific promoter, a Cre-dependent expression, viral tropism, or an infection of neuronal projections far away from the stimulation site. All of these methods allow unequaled temporo-spatial control of the delivered stimulation (Goshen 2014).

For the last few years, optogenetics has helped to investigate numerous questions concerning learning and memory processes, which is reflected in the substantial number of review articles related to the area: from fear and reward circuits (Nieh et al. 2013) to hippocampal engram cells (Ramirez et al. 2014), from the prefrontal cortex (Riga et al. 2014) to the amygdala (LaLumiere 2014), these are only a few examples of the diversity of the recent interests in this field. However, none of the previous reviews have discussed the contribution of optogenetics in the context of olfaction. Here we focus on the central role of optogenetics for the understanding of how the brain recognizes odors, how it memorizes them and how animals make decisions based on odors they are capable of sensing. Flashes of light can replace odor delivery when experiments require precise control over stimulus delivery. For this, the olfactory sensory neurons that normally detect specific odorant chemicals are genetically modified to become sensitive to light. Furthermore, optogenetics has already helped to investigate the circuits of odor learning and olfactory memory. Finally, thanks to optogenetics, one can now modify olfactory memories simply through light delivery as described below.

## Smelling the light

Unlike other sensory inputs such as light or sound, odors diffuse relatively slowly from their source to the olfactory sensory organ where they activate the olfactory receptor neurons (ORN), usually located in insect sensillum or vertebrate olfactory epithelium. Each ORN codes for only one type of odor receptor (Lledo et al. 2005; Kermen et al. 2013; Wilson 2013). Because the olfactory sensory organ is easy to reach, it is conceivable to use optogenetics to mimic neuronal excitation or inhibition traditionally triggered by odor delivery. The first part of this review is dedicated to explaining, through several examples, how optogenetics can turn light into a “false” odor.

To begin, it is noteworthy that optogenetic tools allow for precise temporal control that is critical for studies of short-term neural processes such as decision-making. For instance, this approach was implemented to study navigational decisions in *Drosophila* larvae (Gepner et al. 2015). The larvae are known to avoid light to increase their survival in the dark but are attracted by some odorant cues such as ethyl acetate (EtAc). It has previously been shown that the brighter the light, the more likely larvae are to start turning away. On the contrary, the more concentrated the EtAc, the less frequently the larvae turn away. Gepner et al. (2015) decided to drive expression of a red light-sensitive ChR2 into the EtAc-responding ORNs. To do so, they used a UAS/GAL4 system: they crossed UAS-ChR2 flies with flies expressing GAL4 under the control of the promoter for the EtAc olfactory receptor. This way, the progeny expressed ChR2 specifically in the EtAc ORNs. Given that larvae cannot detect red light, red flashes would then mimic EtAc presentation while blue light would elicit light-related escape behaviors. Furthermore, since *Drosophila* larvae are transparent, hanging a light above them is sufficient to stimulate the ChR2. The authors placed the larvae in the arena and tracked their movements when blue or red light was switched on and off. They were thereby able to compare two navigational decisions: light avoidance and EtAc attraction. The temporal precision of EtAc-like light delivery allowed the authors to compare their results to different models of navigational computation.

In addition to temporal precision, optogenetics also offers advantages in spatial precision. For example, one can express ChR2 in a particular subset of neurons and stimulate them while investigating the response of other neuron subsets one by one. This was achieved in a recent study that uses insects as a system model (Tabuchi et al. 2013). In insects, ORNs project to a structure called the antennal lobe, where they connect to the projection neurons (PNs). Male moths are able to detect a sex pheromone called bombykol with high sensitivity. The authors wanted to understand how PNs responded to very small bombykol concentrations. In order to get fine control over ORN stimulation, ChR2 expression was driven in ORNs in the same fashion as performed to explore navigational decisions in *Drosophila* larvae (Gepner et al. 2015). Moths were head fixed and the electrical activity of PNs was recorded while ORNs were lit up. In this context, the authors could demonstrate that the timing of ORN stimulation is critical for PNs to respond to low light power—which was equivalent to low bombykol concentrations.

The two first examples investigated olfactory processes in insects. While in fact, optogenetics may be used in various other species, including mammals. Insects and mammals share several neural features regarding their olfactory system. In particular, the equivalent of the insect sensillum is the mammal nose, or more precisely the epithelial tissue located inside the nasal cavity named olfactory epithelium. There, odors stimulate ORNs embodied in the stratified layers of the olfactory epithelium. In mammals, the axon of ORNs projects to the equivalent of the antennal lobe, the main olfactory bulb (OB), where they connect to projecting neurons named mitral/tufted cells (MTs), the equivalent of PNs (Table 1).

**Table 1.**
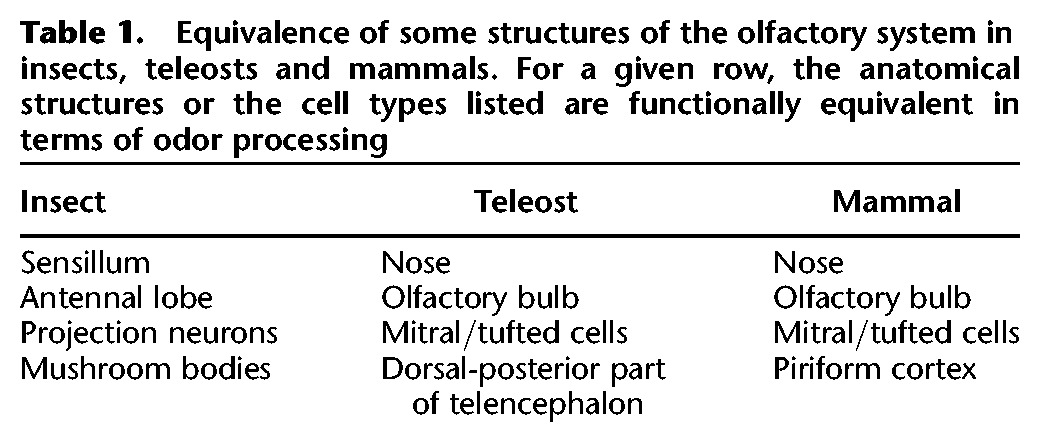
Equivalence of some structures of the olfactory system in insects, teleosts and mammals. For a given row, the anatomical structures or the cell types listed are functionally equivalent in terms of odor processing

In the same vein as explored in insects, Li et al. (2014) used mice in which ChR2 was expressed as a transgene under the control of an ORN-specific promoter. They addressed whether mice could detect different delays between two deliveries of the same odor. Such a question could hardly be answered with real odor delivery, since odors diffuse slowly in the air. Light delivery, again, was useful in this context because of the precise temporal control it offers. However, unlike most insects, mice do not have transparent skin and their brain is isolated in an opaque skull. In order for flashes of light to reach a mammal's brain, several options already exist. For example, one could slice and photostimulate the ex vivo brain while recording neural activity. This is the option chosen by Li et al. (2014): they used the patch-clamp technique to verify that they could initiate MT responses to the stimulation of ORN projections. However, one might argue that brain slices are not ideal for understating neural process involved in behavioral responses. To solve this obstacle, the authors also implanted an optical fiber in the OB, where ORN projections contact MTs. They then performed extracellular recordings of MTs while sending pairs of short laser pulses through the implanted fiber. They were able to show that a fraction of MTs code for between-pair delays through tonic activity. They also demonstrated that mice could actually use the light delays to perform a discrimination task.

Smear et al. (2013) have also used optogenetics to unravel how information from the sensory organ could be sent to the OB. The authors took advantage of the precise molecular organization of the olfactory system to show that a mouse can perceive activation of a single well-defined class of ORN out of the ∼1000 that it possess. To achieve this, Smear et al. manipulated directly a unique ORN type by linking expression of ChR2 to only one odorant receptor (i.e*.*, the *M72* receptor). Using light activation of a subset of ORNs, they demonstrated that mice performed extremely well at detecting stimulation of the M72 glomerulus. This study demonstrated that a mouse can perceive activation of a single ORN type out of its ∼1000. By recording the output OB neurons, this study also showed the intrinsic circuitry of the OB behaves as an amplifier, converting a small sensory input signal into a larger output, changes that could be then detected and decoded by olfactory cortical centers (Smear et al. 2013).

In sum, optogenetics affords temporal and spatial control over odor stimulation that is simply impossible with a mere odor delivery. This ability to control odor exposure with great resolution has raised new questions, especially with regards to neural computations (i.e*.*, understanding a neuron's transfer function in the OB, defining pattern decorrelation, oscillatory synchrony, and dynamic temporal codes). In addition, optogenetics is a great tool not only because it creates “false” odors that are easily amenable, but it is also a useful approach to understanding learning and memory processes, as described below.

## Revealing olfactory learning and memory processes with light pulses

Defining learning and memory is a risky exercise, especially because the definition can change depending on the area of study that one considers. Following the most consensual definition in neurobiology, learning corresponds to all the changes that occur at the molecular, cellular, and cognitive levels leading to the acquisition of information about the external world. Memory is the knowledge retained after learning (Bailey et al. 1996). Optogenetics is particularly adapted for investigating the physical substrates of the learning and memory mechanisms because, as mentioned previously, it allows temporal as well as spatial precision in terms of neural control. Thus, one can use optogenetics to study learning and memory processes within a particular region of the brain.

Some neurons keep traces of an event by changing their resting membrane potential more or less durably. In 2012, Bundschuh et al. investigated such a phenomenon in the zebrafish OB. Like mammals, teleosts have two nasal cavities at the bottom of which lays the olfactory epithelium (Table 1). ORNs from the epithelium project to the first central relay of the olfactory pathway, the OB, where they form direct synapses with MTs (Kermen et al. 2013). Within the OB circuit, various types of GABAergic interneurons regulate MT activity. A subset of these inhibitory interneurons also releases dopamine (DA), which can hyperpolarize MTs (Bundschuh et al. 2012). The question that remained to be addressed in the field was the specific function of DA in odor processing. For this purpose, they made DAergic interneurons sensitive to light by the expression of ChR2 to their membrane. Then the authors used the patch-clamp technique to record the electrical activity of MTs on OB slices while flashing light to stimulate the ChR2-expressing DAergic interneurons. The authors found that short pulses of light-induced fast GABAergic inhibitory currents in MTs. However, long pulses of light provoked a slow DA-mediated hyperpolarization of MTs. This mechanism may account for the slow adaptation of OB function. In this study, optogenetics was critical because it allowed the targeting of a unique population of neurons with high specificity. Furthermore, compared with simple electrical stimulation, one can stimulate synchronously numerous ChR2-expressing neurons with each flash of light. As such, this approach increases the chances of exciting a given neuron that actually makes synapses with the recorded neuron.

At the circuit level, memory may be supported by synapse formation as well. An advantage of using viral infection to introduce a *ChR2* gene into neurons is that one can label them at a precise age. This approach has been fertile in studying the functional meaning of adult-born neurons in learning and memory processes. In the adult mouse brain, two regions generate newborn neurons: the dentate gyrus, in the hippocampus, and the subventricular zone (SVZ) of the forebrain. In the SVZ, the neurogenic zone is located throughout the lateral walls of the lateral ventricles. There, once produced, newborn neurons migrate through the rostral migratory stream (RMS) en route to the OB, where they mature mostly into a subtype of GABAergic interneuron called the granule cell (GC) (Lledo et al. 2005). Injecting a *ChR2*-encapsulating virus into the RMS results in labeling a cohort of GCs with the same age, in the same manner as a pulse-chase experiment. A previous study used this approach to understand the modalities of synapse formation of newborn GCs in mice (Bardy et al. 2010). The authors described different characteristics of newborn GC synapses at different ages after full completion of the migratory process. In particular, they recorded neuron activity in OB slices while photostimulating newly formed GCs. They demonstrated that GCs start forming functional synapses while still maturing. However, most synapses are formed one month later and full maturation was reached only several months later.

If optogenetics has been a precious approach adapted to the study of learning and memory processes within a particular region of the brain it has been also useful to investigate how different parts of the brain interact with each other. For instance, it has been a fruitful approach to decipher the role of the orbitofrontal cortex (OFC) in learning and in encoding reward outcomes. The OFC is reciprocally connected with the dorsal raphe nucleus (DRN), the major nucleus of serotoninergic neurons. However, until recently, it was unclear how the DRN modulates the reward activity of the OFC. To address this question, Zhou et al. (2015) have labeled serotoninergic DRN neurons with ChR2 by injecting a *ChR2*-encapsulating virus into the DRN. The ChR2 gene expression was Cre-recombinase dependent. Because the mice chosen for this study expressed Cre-recombinase under the control of a serotonin promoter, the authors were able to specifically interrogate serotoninergic DRN neurons. The authors implanted an optical fiber into the DRN and performed a Pavlovian conditioning task on mice, while recording OFC activity and stimulating the DRN. They demonstrated that DRN photostimulation is sufficient to modulate the neural response of the OFC to reward outcome. They proposed that the DRN modulates the responses of OFC neurons to the expectation of a reward.

These few examples demonstrate how much optogenetics is useful to study the circuits of learning and memory, by offering fine control over neuronal electrical activity. This advantage is used not only to identify neuronal circuits involved in olfactory-driven behaviors, but also to manipulate the strength of olfactory memory.

## Shining light to foster olfactory learning and memory

The *Men in Black* manipulates memories with light. More and more neuroscientists do the same nowadays as optogenetics can be used to increase learning abilities. For instance, Alonso et al. (2012) showed that, under precise conditions, enhancing the activity of newborn GCs with light was sufficient to facilitate odor learning in mice. The authors injected a *ChR2*-encapsulating lentivirus into the RMS of mice, as described earlier by Bardy et al. (2010), so that the light-sensitive ChR2-expressing GCs in the OB were all born postnatally. They then tested the ability of the mice to learn odors, while stimulating the newborn GCs with different frequencies of light and with different delays relative to the odor delivery. The authors found that only light delivery at high frequencies (i.e*.*, 40Hz), when provided synchronously during odor presentation, were able to facilitate odor learning. This experiment also illustrates how crucial the choice of light protocol is because in this study, light was ineffective when the photostimulation was delivered too early, too late, or when the delivery frequency was too low.

Not only have scientists worked on enhancing odor learning, they have also tried to create new odor memories—and with success. A previous study addressed the question of electrical changes in the mouse OB following an initial odor presentation (Patterson et al. 2013). The authors recorded the activity of MTs while presenting odors to awake, head-fixed mice. They were able to demonstrate that a fraction of the MTs maintained their odor-evoked response even after odor cessation. The duration and the strength of these after-odor responses depended on the actual odor presentation. They also disappeared gradually and did not code for odor representation. Therefore, the authors proposed that these after-odor responses were odor afterimages, a form of short-term memory. They then tried to generate such afterimages without odors. To do that, the authors performed the same recordings with transgenic mice, in which ChR2 was expressed as a transgene under the control of a MT-specific promoter. During these recordings, they photostimulated the MTs with an optical fiber placed at the surface of the OB and coupled to a laser. Just like odors, light stimulations were able to generate afterimages, but the behavioral changes of the light-induced memory were not explored.

In 2006, Schroll and colleagues investigated the mechanisms of conditioning learning in *Drosophila* larvae. It was previously shown that DA neurons were involved in aversive learning while octopaminergic (OA) neurons were crucial for appetitive learning. Could the stimulation of DA or OA neurons be sufficient to change the value of an odor? The authors labeled DA or OA neurons with ChR2. They then exposed the larvae to odors while photostimulating them. Light exposure was sufficient to induce aversive reaction to the odors in DA-labeled larvae. In the same fashion, light exposure induced attractive behavior to the same odors in OA-labeled larvae. Therefore, optogenetics allowed the authors to investigate conditioning learning in freely moving larvae. In both cases, light exposure changed the value of a neutral odor.

To put it all in a nutshell, optogenetics has been, and will be, a powerful tool to manipulate brain circuits in general, and those involved in olfactory learning and memory, in particular. With this tool in hands, it is possible to decipher the neuronal circuitry underlying these two processes, but also to enhance learning abilities, as well as creating de novo memories or erase unpleasant ones.

## Conclusions

In general, odor detection relies on the activation of several different ORNs, each of them responding to precise molecular features (Lledo et al. 2005). Therefore, the study of odor processes requires fine control over which ORNs are targeted and when they are stimulated. For this reason, in less than a decade, optogenetics has become a tool broadly adopted in every field of neuroscience, including the study of sensory systems. Compared with odor delivery, optogenetics can provide incredible accuracy: one can target individual neurons and precisely control the timing of stimulation in order to mimic odor delivery. The strength of optogenetics may also be applied to analyze and describe neuronal circuits underlying olfactory information processing as well as learning and memory. From the memory traces of single neurons to the dialogue between different brain areas during learning processes, numerous questions can be addressed with optogenetics. In fact, because optogenetics can be combined with various other experimental approaches including patch-clamp technique, in vivo electrophysiological recordings, calcium imaging, and behavioral assays, we are just starting to recognize the power of this technique when tempting to establish a causal link between neural circuit activity and olfactory behaviors.

From all of the studies discussed in this review article (Table 2), two major questions can be deduced that anyone planning to use optogenetics should address: (1) how can one target the right neuron population? (2) How can one access the targeted neurons with light? All the methods for targeting neurons may be grouped into two categories: the *ChR2* gene is already in the organism, or it needs to be injected at the inception of the experiment. Thus, the spatial specificity of optogenetics may be given by a judicious choice of promoter driving the ChR2 expression, a precise injection site for the *ChR2* construct, or a combination of both.

**Table 2.**
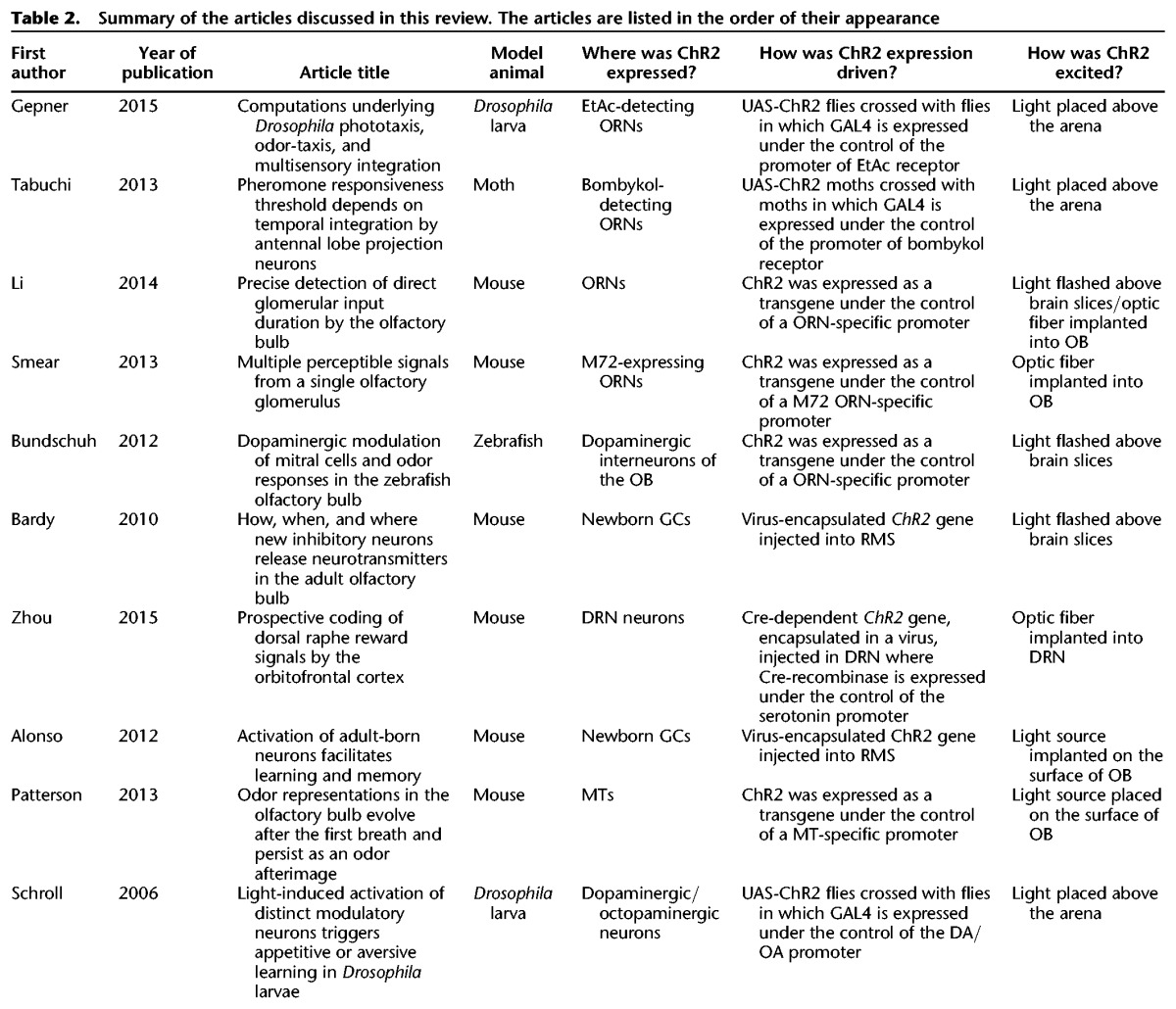
Summary of the articles discussed in this review. The articles are listed in the order of their appearance

Lighting up the ChR2-expressing neurons can be challenging to a greater or lesser degree depending on the animal model. Most insects are transparent enough to let the light transverse their exoskeleton to the brain. In the same manner, zebrafish embryos are transparent and do not require any specific light setup. Usually, scientists working on insects or zebrafish photostimulate the ChR2-expressing neurons by lighting up the whole organism with a light source placed above or below the experimental setup. However, in behavioral assays with several organisms tracked at the same time, such simplicity becomes a weakness for one who wants to stimulate animals selectively. A new method called Automated Laser Tracking and Optogenetic Manipulation System (ALTOMS) solves this problem: a computer tracks each individual in real time. It also controls a laser that can fire a flash of light to any single animal when needed (see Wu et al. 2014). Although this method has been developed for *Drosophila*, surely it could be successfully applied to more organisms.

On the contrary, mammals have pigmented skin and opaque skulls. Scientists have two main options to photostimulate mammal brains: put the light on the surface of the brain or have the light source implanted. In all cases, it requires invasive surgery and the implantation of the light source onto the animal's head. The deeper the region of interest, the more invasive the surgery will be. One recent answer to this problem is the use of red-shifted opsins, such as the new inhibitory opsin called Jaws (Chuong et al. 2014). Another solution is to have the light produced directly in the region of interest through a chemical reaction. Recently, a team coexpressed luciferase and an inhibitory opsin in the mouse striatum. They were able to show that luciferin injection into the striatum could activate the opsins and reduce the striatum activity (Land et al. 2014).

With the exception of the two last articles cited above (Chuong et al. 2014; Land et al. 2014), one may notice that none of the studies reviewed here use inhibitory opsins. The reason is simple: while activating 5%–10% of a given neuronal population could provide behavioral changes (through a gain of function), it is more difficult to detect behavioral alterations when reducing (or inhibiting) a small fraction of a given population. Nevertheless, both approaches are complementary and need to be implemented. While excitatory channels can be used to show how a particular subset of neurons is sufficient to elicit a certain behavior, inhibitory channels can provide clues about the necessity of some neurons for a particular behavior.

A good example of this strategy is a recent study by Stubblefield et al. (2013). The superior colliculus (SC), one in each hemisphere, is known to play a role in orienting the motor outputs to the right or the left based on sensory information. At the time of the study, it was known from in-slice studies that SC contained a majority of excitatory, projecting neurons and a few inhibitory interneurons. It was thought, but not proven, that excitatory neurons were responsible for orienting movements toward the contralateral side of the SC, while inhibitory neurons-guided movements toward the ipsilateral side. The authors drove the expression of ChR2 or a halorhodopsin into one of the SC of mice through a viral injection. Since the vast majority of the SC neurons are excitatory, they assumed light stimulations would affect mainly the excitatory neurons. The mice were trained on a two-alternative movement task, in which they had to get a reward by poking their nose into a hole to the right or left of the odor presentation, depending on the odor exposure. When the authors tested the mice after training, they show that exciting the SC with ChR2 biased mouse decisions toward the contralateral side. On the contrary, inhibiting the SC with halorhodopsin biased the mice toward the ipsilateral side. Therefore, the activation of the SC appears not only sufficient, but also necessary, to drive the mouse decision toward the contralateral side. This example shows how useful inhibitory light-activated channels can be, and how one could easily take advantage of them for the study of olfactory learning and memory processes.

Last but not least, when using optogenetic stimulations, one should always keep in mind the limitations of the tool. First of all, the way neurons are stimulated can drastically change the way they respond and, eventually, affect the behavior. The study by Alonso et al. (2012) discussed above is a great example: not only did light have to be delivered at 40 Hz, but it also needed to be synchronously delivered with odor presentations, to enhance olfactory learning. Furthermore, the way animals sniff influences the way odors are presented to the olfactory epithelium. The duration of the odor presentation varies with the sniffing rate, and the odor concentration in the nasal cavity varies within a single sniffing cycle (Scott 2006). As a consequence, one cannot accurately mimic an odor presentation with light stimulations at a constant rate: the OB stimulations caused by odor presentations are much more complex. Finally, due to the relative transparency of the brain and the size of the light source, a single light pulse is likely to stimulate numerous ChR2-expressing neurons at the same time (Alonso et al. 2012). Beyond the fact that this synchrony may have no biological significance, it can affect the neuronal readout. In fact, the synchrony of the neuronal activity increases the synchrony of noisy signals, leading to a decrease in the signal-to-noise ratio and, ultimately, a loss of information (Uchida et al. 2006).

To summarize, compared with odor presentation, optogenetic stimulation carries limitations, such as the difficulty to find the right pattern of light stimulation, the biological significance of this pattern compared with a real odor presentation, and the induced supernatural synchrony. Notwithstanding these limitations, the advent of optogenetics has allowed the raising—and the response to—numerous questions regarding odor learning processes and memory pathways, thanks to its spatial and temporal capacities. Since optogenetic techniques are relatively recent and constantly evolving, surely this will bring new “light” to how we smell, learn, and remember.

## References

[GRIMAUDLM041269C1] Alonso M, Lepousez G, Sebastien W, Bardy C, Gabellec MM, Torquet N, Lledo PM. 2012 Activation of adult-born neurons facilitates learning and memory. Nat Neurosci 15: 897–904. 22581183 10.1038/nn.3108

[GRIMAUDLM041269C2] Bailey CH, Bartsch D, Kandel ER. 1996 Toward a molecular definition of long-term memory storage. Proc Natl Acad Sci 93: 13445–13452. 8942955 10.1073/pnas.93.24.13445 PMC33629

[GRIMAUDLM041269C3] Bardy C, Alonso M, Bouthour W, Lledo PM. 2010 How, when, and where new inhibitory neurons release neurotransmitters in the adult olfactory bulb. J Neurosci 30: 17023–17034. 21159972 10.1523/JNEUROSCI.4543-10.2010 PMC6634902

[GRIMAUDLM041269C4] Boyden ES, Zhang F, Bamberg E, Nagel G, Deisseroth K. 2005 Millisecond-timescale, genetically targeted optical control of neural activity. Nat Neurosci 8: 1263–1268. 16116447 10.1038/nn1525

[GRIMAUDLM041269C5] Bundschuh ST, Zhu P, Schärer YP, Friedrich RW. 2012 Dopaminergic modulation of mitral cells and odor responses in the zebrafish olfactory bulb. J Neurosci 32: 6830–6840. 22593052 10.1523/JNEUROSCI.6026-11.2012 PMC6622199

[GRIMAUDLM041269C6] Chuong AS, Miri ML, Busskamp V, Matthews GA, Acker LC, Sørensen AT, Young A, Klapoetke NC, Henninger MA, Kodandaramaiah SB, 2014 Noninvasive optical inhibition with a red-shifted microbial rhodopsin. Nat Neurosci 17: 1123–1129. 24997763 10.1038/nn.3752 PMC4184214

[GRIMAUDLM041269C7] Crick F. 1999 The impact of molecular biology on neuroscience. Philos Trans R Soc Lond B Biol Sci 354: 2021–2025. 10670022 10.1098/rstb.1999.0541 PMC1692710

[GRIMAUDLM041269C8] Gepner R, Mihovilovic Skanata M, Bernat NM, Kaplow M, Gershow M. 2015 Computations underlying *Drosophila* photo-taxis, odor-taxis, and multi-sensory integration. Elife 4. 10.7554/eLife.06229 PMC4466338 25945916

[GRIMAUDLM041269C9] Goshen I. 2014 The optogenetic revolution in memory research. Trends Neurosci 37: 511–522. 25022518 10.1016/j.tins.2014.06.002

[GRIMAUDLM041269C10] Guru A, Post RJ, Ho YY, Warden MR. 2015 Making sense of optogenetics. Int J Neuropsychopharmacol 18: pyv079. 26209858 10.1093/ijnp/pyv079 PMC4756725

[GRIMAUDLM041269C11] Kermen F, Franco LM, Wyatt C, Yaksi E. 2013 Neural circuits mediating olfactory-driven behavior in fish. Front Neural Circuits 7: 62. 23596397 10.3389/fncir.2013.00062 PMC3622886

[GRIMAUDLM041269C12] LaLumiere RT. 2014 Optogenetic dissection of amygdala functioning. Front Behav Neurosci 8: 107. 24723867 10.3389/fnbeh.2014.00107 PMC3972463

[GRIMAUDLM041269C13] Land BB, Brayton CE, Furman KE, Lapalombara Z, Dileone RJ. 2014 Optogenetic inhibition of neurons by internal light production. Front Behav Neurosci 8: 108. 24744708 10.3389/fnbeh.2014.00108 PMC3978322

[GRIMAUDLM041269C14] Li A, Gire DH, Bozza T, Restrepo D. 2014 Precise detection of direct glomerular input duration by the olfactory bulb. J Neurosci 34: 16058–16064. 25429146 10.1523/JNEUROSCI.3382-14.2014 PMC4244471

[GRIMAUDLM041269C15] Lledo PM, Gheusi G, Vincent JD. 2005 Information processing in the mammalian olfactory system. Physiol Rev 85: 281–317. 15618482 10.1152/physrev.00008.2004

[GRIMAUDLM041269C16] Nieh EH, Kim SY, Namburi P, Tye KM. 2013 Optogenetic dissection of neural circuits underlying emotional valence and motivated behaviors. Brain Res 1511: 73–92. 23142759 10.1016/j.brainres.2012.11.001 PMC4099056

[GRIMAUDLM041269C17] Patterson MA, Lagier S, Carleton A. 2013 Odor representations in the olfactory bulb evolve after the first breath and persist as an odor afterimage. Proc Natl Acad Sci 110: E3340–E3349. 23918364 10.1073/pnas.1303873110 PMC3761593

[GRIMAUDLM041269C18] Ramirez S, Tonegawa S, Liu X. 2014 Identification and optogenetic manipulation of memory engrams in the hippocampus. Front Behav Neurosci 7: 226. 24478647 10.3389/fnbeh.2013.00226 PMC3894458

[GRIMAUDLM041269C19] Riga D, Matos MR, Glas A, Smit AB, Spijker S, Van den Oever MC. 2014 Optogenetic dissection of medial prefrontal cortex circuitry. Front Syst Neurosci 8: 230. 25538574 10.3389/fnsys.2014.00230 PMC4260491

[GRIMAUDLM041269C20] Schroll C, Riemensperger T, Bucher D, Ehmer J, Völler T, Erbguth K, Gerber B, Hendel T, Nagel G, Buchner E, 2006 Light-induced activation of distinct modulatory neurons triggers appetitive or aversive learning in *Drosophila* larvae. Curr Biol 16: 1741–1747. 16950113 10.1016/j.cub.2006.07.023

[GRIMAUDLM041269C21] Scott JW. 2006 Sniffing and spatiotemporal coding in olfaction. Chem Senses 31: 119–130. 16354743 10.1093/chemse/bjj013 PMC2229829

[GRIMAUDLM041269C22] Sineshchekov OA, Jung KH, Spudich JL. 2002 Two rhodopsins mediate phototaxis to low- and high-intensity light in *Chlamydomonas reinhardtii*. Proc Natl Acad Sci 99: 8689–8694. 12060707 10.1073/pnas.122243399 PMC124360

[GRIMAUDLM041269C23] Smear M, Resulaj A, Zhang J, Bozza T, Rinberg D. 2013 Multiple perceptible signals from a single olfactory glomerulus. Nat Neurosci 16: 1687–1691. 24056698 10.1038/nn.3519 PMC13445371

[GRIMAUDLM041269C24] Stubblefield EA, Costabile JD, Felsen G. 2013 Optogenetic investigation of the role of the superior colliculus in orienting movements. Behav Brain Res 255: 55–63. 23643689 10.1016/j.bbr.2013.04.040 PMC3796036

[GRIMAUDLM041269C25] Tabuchi M, Sakurai T, Mitsuno H, Namiki S, Minegishi R, Shiotsuki T, Uchino K, Sezutsu H, Tamura T, Haupt SS, 2013 Pheromone responsiveness threshold depends on temporal integration by antennal lobe projection neurons. Proc Natl Acad Sci 110: 15455–15460. 24006366 10.1073/pnas.1313707110 PMC3780863

[GRIMAUDLM041269C26] Wilson RI. 2013 Early olfactory processing in *Drosophila*: mechanisms and principles. Annu Rev Neurosci 36: 217–241. 23841839 10.1146/annurev-neuro-062111-150533 PMC3933953

[GRIMAUDLM041269C27] Wu MC, Chu LA, Hsiao PY, Lin YY, Chi CC, Liu TH, Fu CC, Chiang AS. 2014 Optogenetic control of selective neural activity in multiple freely moving *Drosophila* adults. Proc Natl Acad Sci 111: 5367–5372. 24706830 10.1073/pnas.1400997111 PMC3986155

[GRIMAUDLM041269C28] Uchida N, Kepecs A, Mainen ZF. 2006 Seeing at a glance, smelling in a whiff: rapid forms of perceptual decision making. Nat Rev Neurosci 7: 485–491. 16715056 10.1038/nrn1933

[GRIMAUDLM041269C29] Zemelman BV, Lee GA, Ng M, Miesenbock G. 2002 Selective photostimulation of genetically charged neurons. Neuron 33: 15–22. 11779476 10.1016/s0896-6273(01)00574-8

[GRIMAUDLM041269C30] Zhou J, Jia C, Feng Q, Bao J, Luo M. 2015 Prospective coding of dorsal raphe reward signals by the orbitofrontal cortex. J Neurosci 35: 2717–2730. 25673861 10.1523/JNEUROSCI.4017-14.2015 PMC6605606

